# Three-Dimensional Measurement for Specular Reflection Surface Based on Reflection Component Separation and Priority Region Filling Theory

**DOI:** 10.3390/s17010215

**Published:** 2017-01-23

**Authors:** Xiaoming Sun, Ye Liu, Xiaoyang Yu, Haibin Wu, Ning Zhang

**Affiliations:** The Higher Educational Key Laboratory for Measuring & Control Technology and Instrumentations, Harbin University of Science and Technology, Harbin 150080, China; yiqvtonggong@outlook.com (Y.L.); woo@hrbust.edu.cn (H.W.); zhnk2016@126.com (N.Z.)

**Keywords:** three-dimensional measurement, reflection component separation, specular highlight removal, priority region filling theory

## Abstract

Due to the strong reflection property of materials with smooth surfaces like ceramic and metal, it will cause saturation and the highlight phenomenon in the image when taking pictures of those materials. In order to solve this problem, a new algorithm which is based on reflection component separation (RCS) and priority region filling theory is designed. Firstly, the specular pixels in the image are found by comparing the pixel parameters. Then, the reflection components are separated and processed. However, for ceramic, metal and other objects with strong specular highlight, RCS theory will change color information of highlight pixels due to larger specular reflection component. In this situation, priority region filling theory was used to restore the color information. Finally, we implement 3D experiments on objects with strong reflecting surfaces like ceramic plate, ceramic bottle, marble pot and yellow plate. Experimental results show that, with the proposed method, the highlight caused by the strong reflecting surface can be well suppressed. The highlight pixel number of ceramic plate, ceramic bottle, marble pot and yellow plate, is decreased by 43.8 times, 41.4 times, 33.0 times, and 10.1 times. Three-dimensional reconstruction results show that highlight areas were significantly reduced.

## 1. Introduction

Structured light (SL) vision measurement has drawn much attention due to its potential for three-dimensional (3D) applications to diverse areas, such as re-engineering, 3D games, industrial inspection, object recognition, and clothing design, to name only a few [1]. In the field of structured light, phase calculation-based fringe projection techniques are actively studied in academia and widely applied in industry because of the advantages of non-contact operation, full-field acquisition, high accuracy, fast data processing and low cost [2,3,4]. However, in virtually any real-world application, especially in industry, there are large numbers of specular objects need to be measured. Current fringe projection methods are unable to cope with scene regions that produce strong highlights due to specular reflection. Highlights can cause the camera saturation, change the gray distribution of the laser stripe, and influence the accuracy of stripe center extraction. Highlight removal remains a major challenge for SL vision measurement area [5].

Many methods have been developed for separating specular and diffuse reflection components. Shafer proposed dichromatic reflection model to separate reflection components [6]. Klinker et al. found T-shaped color distribution features of diffuse and highlights in the RGB color space to detect and remove highlights, but this T-shaped color distribution is sensitive to noise [7]. Mallick et al. proposed a highlight removal method on the assumption that the color of light source was known, which can avoid segmentation algorithm of image [8]. Kokku et al. proposed template feature extraction method [9], but it is not useful for objects with complex features or no features. Yang [10] used a homomorphic filtering algorithm to remove highlights, which is based on partial differential equation. Chai put forward a method [11] based on a frequency domain filter. This method compared highlights and diffuse light frequency spectrum to make a frequency domain filter for removing highlights, but it was only applicable to the situation in which curvature change was not obvious.

For the above-mentioned methods, segmentation needs to be performed, which limits the technique’s effectiveness. Wolff and Boult [12] used the polarization method to separate the reflection components. A key observation of their method is that, for most incident angles, specular reflections become polarized, while diffuse reflections are basically unpolarized. Nayar et al. [13] extended this work by considering colors instead of using the polarizing filter alone. Sohn [14] analyzed the relationship between specular objects and polarized reflectivity. Tsuru [15] used elliptical polarization method to measure 3D specular objects. However, the polarization method needed more polaroids, which increased the complexity of measurement. In addition, if an experiment used an unpolarized light source, it needs lots of images in different polarized directions.

Rogerio Feris used multi-light sources to reduce the highlight region, but the highlight region area cannot be completely removed [16]. Y. Liu [17] used multiple images taken under different light sources to remove the highlight region. Qian [18] and Harding [19] took the same image from different angles to reduce highlights, but this method can create a complex splicing problem. Sato and Ikeuchi et al. [20,21] took a series of images by moving the light source, and analyzed the color information of multiple images to remove the specularities. G. H. Liu [22] used a multiple exposure method and adjusted exposure time to make the specularities unsaturated, but it can bring fringe center offset phenomenon, which cannot meet measurement accuracy requirements. Asundi [23] used the spray method to change the reflection characteristics of the metal surface to eliminate the specularities, but this method had certain corrosion to the blade surface. Jiang [24] used a spherical light source to measure the surface with strong reflection characteristics. In 2007, Guo [25] used a moving diffuse light source to reduce highlights. In 2012, the diffuser was applied to the strong reflection measurement field by Nayar [5]. In 2014, Sills [26] used the high power light emitting diode to irradiate surface of strong reflection directly.

Through the above analysis, dichromatic reflection model is suitable for nonconductive material, but not for ceramics and metal surfaces. The polarization method is easy to cause the camera’s saturation when incident angle is close to 90 degrees, and it only works well for dielectric specular reflections as opposed to metallic specular reflections. Multi-light sources and the multi-exposure method still has overlapped parts of highlight regions. Some image processing methods require multiple images taken under specific conditions, but, for many applications, using multiple images is impractical. In addition, a single input image method requires complex color segmentation to deal with multi-colored images.

For this problem, we proposed a specular highlight removal method based on reflection component separation (RCS) and priority pixel filling theory, which does not depend on either polarization or image segmentation. This method is based on color information completely without requiring any geometrical information of the object surface. Reflection component separation can reduce the intensity of highlight area, and make the specular reflection component of the object decrease. However, for ceramic, metal and other objects with strong specular highlight, RCS theory will change color information of highlight pixel due to larger specular reflection component. In this situation, the priority pixel filling theory was used to restore the color information. Finally, the proposed method was applied to reconstruct ceramic surfaces with strong specular highlights.

## 2. Specular Highlight Removal

Reflection component separation theory is proposed by Robby T. Tan, which is based on Shafer’s dichromatic reflection model [27]. In this theory, firstly, in order to reduce the pixel intensity, chroma values of two adjacent pixels are compared and the larger maximum chroma value is assigned to the smaller one. Then, each pair of adjacent pixels in the whole image are compared to reduce the image intensity.

### 2.1. Reflection Model

**Figure 1:**
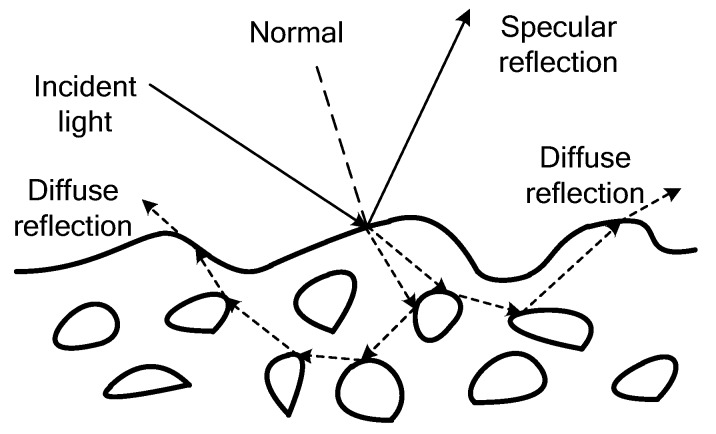
Dichromatic reflection model.

As shown in Figure 1, the medium comprises the bulk of the matter and is approximately transparent in general, while the pigments selectively absorb the light and scatter it by reflection and refraction. Most inhomogeneous objects exhibit both diffuse and specular reflections. Considering these two reflection components, Shafer [6] proposed this dichromatic reflection components, which states that reflected lights of inhomogeneous objects are a linear combination of diffuse and specular reflection components.

As a result, each pixel of the image taken by the Charge Coupled Device (CCD) is a linear combination of diffuse component and specular component, which can be described as

(1) $$I\left(x\right)=\alpha \left(x\right)\int_{\Omega }S(\lambda ,x)B\left(\lambda \right)Q\left(\lambda \right)d\lambda +\beta \left(x\right)\int_{\Omega }B(\lambda )Q\left(\lambda \right)d\lambda ,$$

where I(x) is the color vector of image intensity, and the spatial parameter, and *x* is the two-dimensional image coordinates. α(x) and β(x) are the weighting factors for diffuse and specular reflections, respectively. S(λ,x) is the diffuse spectral reflectance function and the parameter *λ* represents wavelength of the light spectrum, while B(λ) is the spectral power distribution function of illumination. Q(λ) is the three element-vector of sensor sensitivity, and the integration is done over the visible spectrum Ω. For the sake of simplicity, Equation (1) can be written as:

(2) I(x)=α(x)D(x)+β(x)G,

where $D\left(x\right)=\int_{\Omega }S(\lambda ,x)B\left(\lambda \right)Q\left(\lambda \right)d\lambda$ and $G=\int_{\Omega }B(\lambda )Q\left(\lambda \right)d\lambda$. α(x)D(x) denotes the diffuse reflection component, while β(x)G represents the specular reflection component.

In the dichromatic reflection model, we also need to know the chroma of the image, which is defined as follows:

(3) $$\sigma \left(x\right)=\frac{I(x)}{I_{r}\left(x\right)+I_{g}\left(x\right)+I_{b}\left(x\right)},$$

where $\sigma =\left\{\sigma_{r},\sigma_{g},\sigma_{b}\right\}$.

When only the diffuse reflection component is contained in the pixel β(x)=0, the chroma value is independent of the diffuse reflection weighting factor α(x). Therefore, the diffuse reflection chroma expression is:

(4) $$\Lambda \left(x\right)=\frac{D(x)}{D_{r}\left(x\right)+D_{g}\left(x\right)+D_{b}\left(x\right)},$$

where $\Lambda =\{\Lambda_{r},\Lambda_{g},\Lambda_{b}\}$. In the same way, when the pixels only have specular reflection component α(x)=0, the chroma value of the pixel is independent of the specular reflection factor β(x). We call this specular chroma with the definition:

(5) $$\Gamma =\frac{G}{G_{r}+G_{g}+G_{b}},$$

where $\Gamma =\{\Gamma_{r},\Gamma_{g},\Gamma_{b}\}$. Consequently, with regards to Equations (4) and (5), Equation (2) can be written in terms of chromaticity:

(6) $$I\left(x\right)=m_{d}\left(x\right)\Lambda \left(x\right)+m_{s}\left(x\right)\Gamma ,$$

where $m_{d}\left(x\right)=\alpha \left(x\right)\left[D_{r}\left(x\right)+D_{g}\left(x\right)+D_{b}\left(x\right)\right]$, $m_{s}\left(x\right)=\beta \left(x\right)\left(G_{r}+G_{g}+G_{b}\right)$.

### 2.2. Selection of Highlight Pixels

Firstly, we need to normalize the image and get the normalized image P and the specular-free image T. For the normalized image P, the normalized diffuse pixel is expressed as: $I^{\prime }\left(x\right)={m^{\prime }}_{d}\left(x\right)\Lambda^{\prime }\left(x\right)$, if we apply logarithmics on this pixel, the equation becomes:

(7) $$\log\left(I^{\prime }\left(x_{1}\right)\right)=\log\left(m_{d}^{\prime }\left(x_{1}\right)\right)+\log\left(\Lambda^{\prime }\right).$$

Then, we apply differentiation operation on this pixel, the equation becomes:

(8) $$\frac{d}{dx}\log\left(I^{\prime }\left(x_{1}\right)\right)=\frac{d}{dx}\log\left({m_{d}}^{\prime }\left(x_{1}\right)\right),$$

while, for the specular-free image T, we can obtain a corresponding pixel in the specular-free image. We describe it as $\overset{o}{I}\left(x_{1}\right)=m_{d}^{\prime }\left(x_{1}\right)k\overset{o}{\Lambda }$, where *k* and $\overset{o}{\Lambda }$ are independent from the spatial parameter. Using the same operations, the logarithmic image intensity is expressed as:

(9) $$\log\left(\overset{o}{I(x_{1})}\right)=\log\left({m_{d}}^{\prime }\left(x_{1}\right)\right)+\log\left(k\right)+\log\left(\overset{o}{\Lambda }\right).$$

After taking the derivative, we can get to the equation:

(10) $$\frac{d}{dx}\log\left(\overset{o}{I}\left(x_{1}\right)\right)=\frac{d}{dx}\log\left({m_{d}}^{\prime }\left(x_{1}\right)\right).$$

Based on the theory of intensity logarithmic differentiation, we can obtain

(11) $$\Delta \left(x\right)=d\log\left(I^{\prime }\left(x\right)\right)-d\log\left(\overset{o}{I}\left(x\right)\right).$$

If Δ=0, the two-neighboring pixels are diffuse pixels. If Δ≠0, the two-neighboring pixels may be specular reflection pixels or two discontinuous pixels or noise. We can be expressed as

(12) $$\Delta \left(x\right)=\left\{\begin{array}{c} =0:diffuse,\\ \begin{array}{c} \;\\ \neq 0:specular\;\mathrm{or}\;\mathrm{color}\;\mathrm{discontinuity}. \end{array} \end{array}\right.$$

Secondly, we need to determine whether the two pixels are discontinuous pixels. Here, we calculate the chroma difference between the two adjacent pixels in the R and G channels:

(13) $$\Delta_{r}=\sigma_{r}^{\prime }\left(x\right)-\sigma_{r}^{\prime }\left(x-1\right),\Delta_{g}=\sigma_{g}^{\prime }\left(x\right)-\sigma_{g}^{\prime }\left(x-1\right),$$

(14) $$\sigma_{r}^{\prime }=\frac{I_{r}^{\prime }}{I_{r}^{\prime }+I_{g}^{\prime }+I_{b}^{\prime }},\sigma_{g}^{\prime }=\frac{I_{g}^{\prime }}{I_{r}^{\prime }+I_{g}^{\prime }+I_{b}^{\prime }}.$$

When $\Delta_{r}>\bar{R}$ and $\Delta_{g}>\bar{G}$ ( $\bar{R}$ and $\bar{G}$ are constants), these two pixels are discontinuous. Otherwise, they are noise or specular reflection pixels. When two neighboring pixels have the same surface color, their chromaticity difference is small, even for specular pixels. Thus, we define $\bar{R}=\bar{G}=0.1$.

After the steps above, we also need to judge if the two pixels are specular reflection pixels or noise. Because the maximum chroma of the noise pixel is a constant value, the maximum chroma of the specular reflection pixel must be unequal. Therefore, we only need to determine if the two pixels chrominance maximum are equal. If they are the same, the two pixels are noise. Otherwise, at least one of them must be a specular pixel. Then, the same operation is done for all pixels iteratively. Finally, we will get the specular reflection pixels of the whole image.

### 2.3. Specular Reflection Component Removal Theory

**Figure 2:**
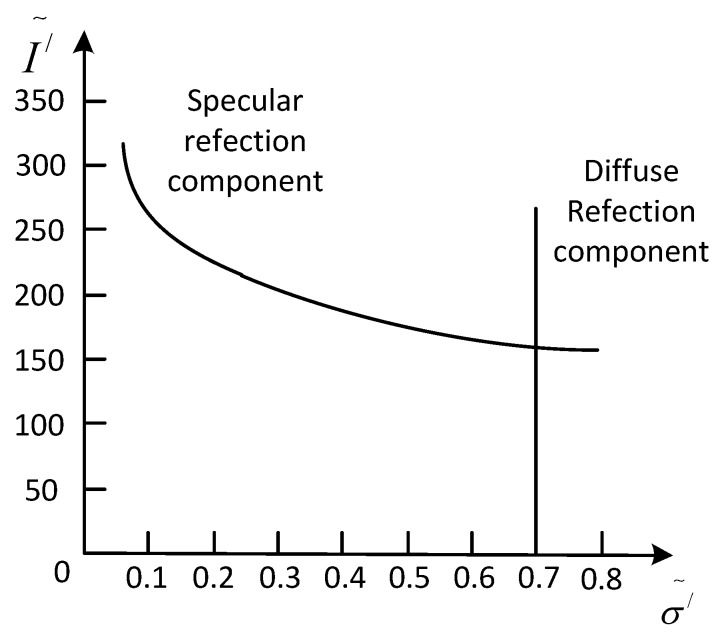
Relationship between diffuse and specular reflection components.

Figure 2 shows the relationship between diffuse and specular reflection components. The maximum chromaticity can be written as: ${\tilde{\sigma }}^{\prime }\left(x\right)=\frac{\max\left({I_{r}}^{\prime }\left(x\right),{I_{g}}^{\prime }\left(x\right),{I_{b}}^{\prime }\left(x\right)\right)}{{I_{r}}^{\prime }\left(x\right)+{I_{g}}^{\prime }\left(x\right)+{I_{b}}^{\prime }\left(x\right)}$, where $\left\{I_{r}^{\prime }\left(x\right),I_{g}^{\prime }\left(x\right),I_{b}^{\prime }\left(x\right)\right\}$ are obtained from a normalized image. For this maximum chromaticity intensity space, with its *x*-axes representing ${\tilde{\sigma }}^{\prime }$ and its *y*-axes representing ${\tilde{I}}^{\prime }$, with ${\tilde{I}}^{\prime }=\max\left(I_{r}^{\prime },I_{g}^{\prime },I_{b}^{\prime }\right)$, the intensity of the specular component is always larger than diffuse components.

The maximum chroma of the diffuse reflection component is not changed with the change of image intensity. However, the maximum chroma of the specular reflection component changes with the intensity of the image. The intensity of the specular reflection component decreases with the increase of the maximum chroma. Finally, the specular component will intersect with the diffuse component at one point. It means that only increasing the maximum chroma of the pixel points is needed, and the specular reflection component can be reduced.

**Figure 3:**
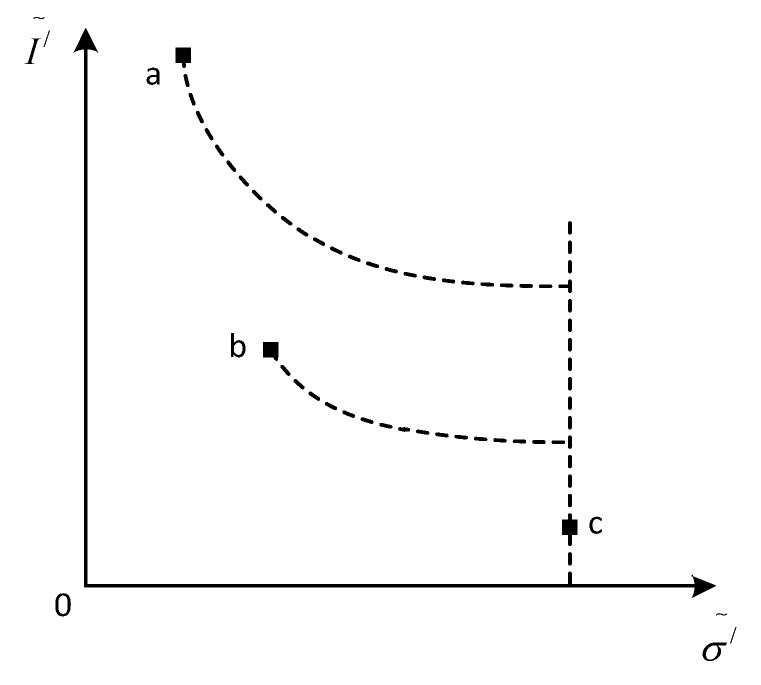
Maximum chroma space.

As shown in Figure 3, we choose three adjacent pixels in the image and defined them as a, b, and c. The specular reflection component of point a is the largest and the diffuse reflection component of point c is the largest, while point b is located in the middle of a and c.

**Figure 4:**
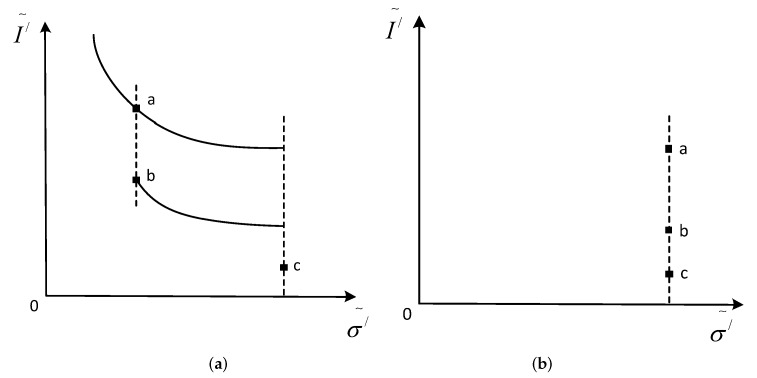
Reflection component separation. (**a**) gives the maximum chroma of b to a; and (**b**) gives the maximum chroma of c to b.

Firstly, the maximum chromas of pixel a and pixel b are compared, and the greater the maximum chroma intensity, the weaker the specular reflection intensity. We can see from Figure 3 that the maximum chroma of point a is less than that of point b. Now, let the maximum chroma of point a be equal to that of b. Then, the specular reflection intensity of point a will be reduced, as shown in Figure 4a. Secondly, we compare the maximum chroma of b and c, and let the maximum chroma of b be equal to that of c. Then, the specular reflection intensity of b will decrease. While the maximum chroma of a is equal to the maximum chroma of b, the specular reflection component of a can be reduced. By analogy, we take iteration for all pixels of surrounding specular reflection area, and the specular reflection component of all pixels will decrease, so as to achieve the purpose of highlight removal.

## 3. Highlight Region Inpainting Based on Priority Theory

**Figure 5:**
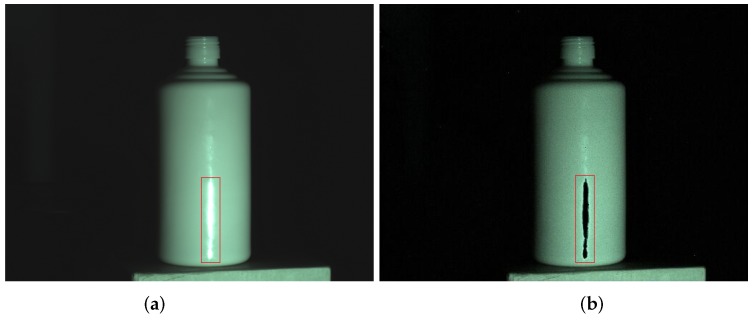
Ceramic bottle processed by specular reflection component removal method. (**a**) input image of ceramic bottle; and (**b**) processed ceramic bottle.

For some strongly reflective surfaces, such as ceramic, glass and metal surfaces, the highlight area is large, as shown in Figure 5a. After the specular reflection component removal method, as shown in Figure 5b, the highlight part has been removed, but the color information is lost and the highlight part becomes black. The reason is that the intensity of the specular reflection component is too large. As we know, specular reflection separation theory is used to subtract the specular reflection component from the pixel and obtain the diffuse reflection component. If the specular reflection component is too large, which almost occupies the whole pixel, the diffuse reflection component is negligible. Therefore, pixels of the highlight area will become black.

### 3.1. Principal of Region Filling Method Based on Priority Theory

**Figure 6:**
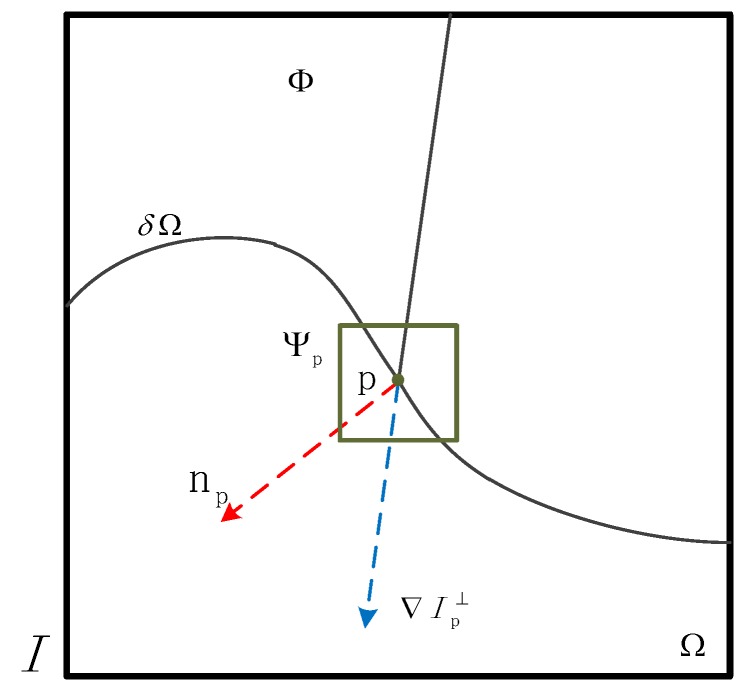
Image annotation.

Figure 6 is a notation diagram of a region-filling algorithm [28], the target region to be filled is indicated by Ω, and its contour is denoted as δΩ, which is the boundary of the area Ω and the source region Φ. Φ remains fixed and provides samples used in Ω. $\psi_{p}$ is a sampling window, and its size can be changed according to the different images. The point P is the center point of patch $\psi_{p}$, for some p∈δΩ, and its priority $P\left(p\right)$ is defined as:

(15) $$P\left(p\right)=C\left(p\right)D\left(p\right),$$

where the $C\left(p\right)$ is the confidence term, and $D\left(p\right)$ is the data term. They show the continuity of the boundary point, which are defined as follows:

(16) $$C\left(p\right)=\frac{\sum_{q\in \psi_{p}\cap \Omega }C\left(q\right)}{\left|\psi_{p}\right|},\;\;\;\;\;\;\;\;\;\;\;D\left(p\right)=\frac{\left|\nabla I_{p}^{⊥}\cdot n_{p}\right|}{\alpha },$$

where $\left|\psi_{p}\right|$ is the area of the $\psi_{p}$, *α* is the normalized factor, and ⊥ is the operation of the orthogonal.

After calculation of priority $P\left(p\right)$, the patch $\psi_{\hat{p}}$ with highest priority is found. We then should find the module $\psi_{\hat{q}}$ that is the most similar to the module $\psi_{\hat{p}}$ in all modules. The $\psi_{\hat{q}}$ should meet the following equation:

(17) $$\psi_{\hat{q}}=\arg\min_{\psi_{\hat{q}}\in \Phi }d\left(\psi_{\hat{p}},\psi_{\hat{q}}\right),$$

where the distance $d\left(\psi_{\hat{p}},\psi_{\hat{q}}\right)$ is defined as the sum of squared differences of the already filled pixels in the two patches.

After the eligible module $\psi_{\hat{q}}$ was found, each pixel in sampling module $\psi_{\hat{p}}$ was filled from its corresponding position inside $\psi_{\hat{q}}$. With the change in pixel of sampling module, priority will also be changed, so the priority should be calculated again. The detailed description is shown in Section 3.2.

### 3.2. Highlight Region Filling Algorithm

We now proceed with the details of our algorithm. The detailed algorithm description is shown in Algorithm 1.
**Algorithm 1. Highlight region filling algorithm**Selected Ω and defined Φ = *I* − ΩDefine *ψ_p_*, and compute *P* (*p*)**(WHILE)** the area Ω has not been completely processed   Find highest priority patch $\psi_{\hat{p}}$ in Φ, and find module $\psi_{\hat{q}}$   Copy data from $\psi_{\hat{q}}$ to $\psi_{\hat{p}}$ in Φ   Updata *C* (*p*)


## 4. Experiment

### 4.1. System Introduction

**Figure 7:**
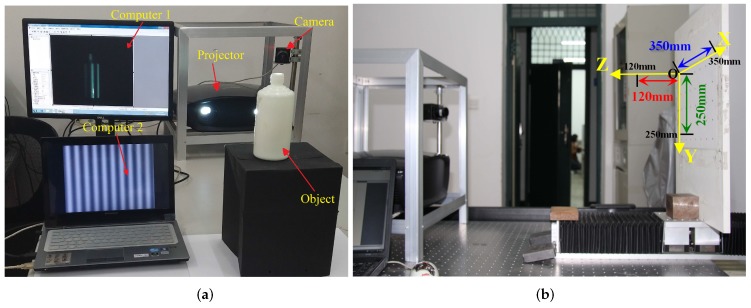
System structure and measuring range. (**a**) system structure; and (**b**) measuring range.

Figure 7 shows the system structure and measuring range. As shown in Figure 7a, the experimental measurement system consists of an industrial camera (DH-HV3151UC, Daheng Image, 2048 × 1536, China Daheng Group, Inc., Beijing, China), a projector (InFocus In82, 1024 × 768, Infocus Visual Digital Technology (Shenzhen) Co., Shenzhen, China) and two computers. After the calibration, stereo rectification, matching and disparity calculation, we can obtain the 3D information of the space points. The separations were processed by Inter Xeon CPU E5-2620 with 16 GB RAM memory. Figure 7b shows measuring range, where *O* is the origin of the measured space, and the measuring range in the *x*-, *y*- and *z*-direction is, respectively, 350 mm, 250 mm and 120 mm.

### 4.2. Visual Comparisons

In the experiment, the ceramic bottle, ceramic plate, marble pot and yellow plate were selected as the measured objects, which have strong reflection characteristics. Then, these images were processed by our proposed method in this paper, and processed images and original images were compared and analyzed.

**Figure 8:**
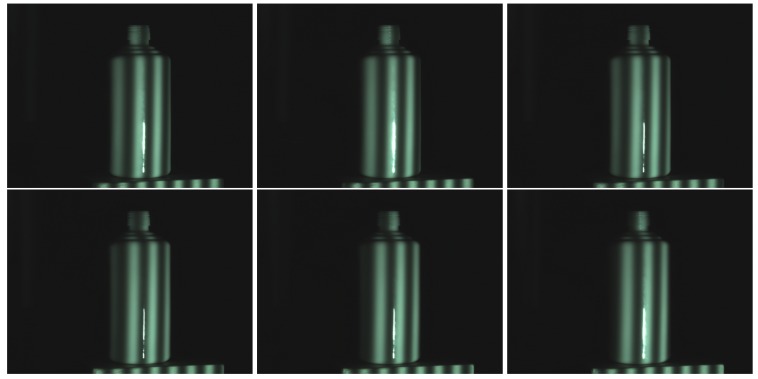
Ceramic bottle influenced by highlight.

**Figure 9:**
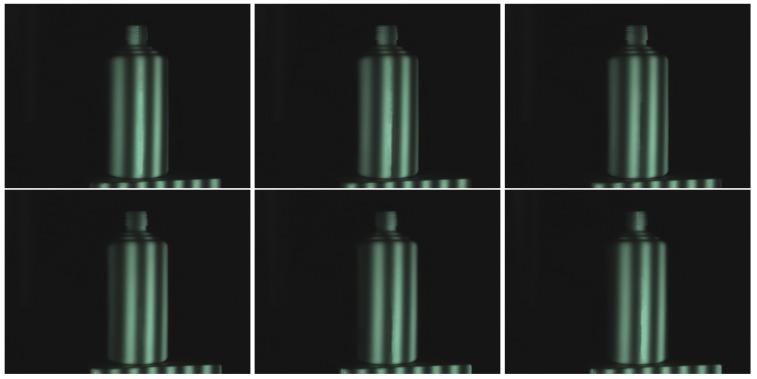
Processed results of ceramic bottle with our method.

**Figure 10:**
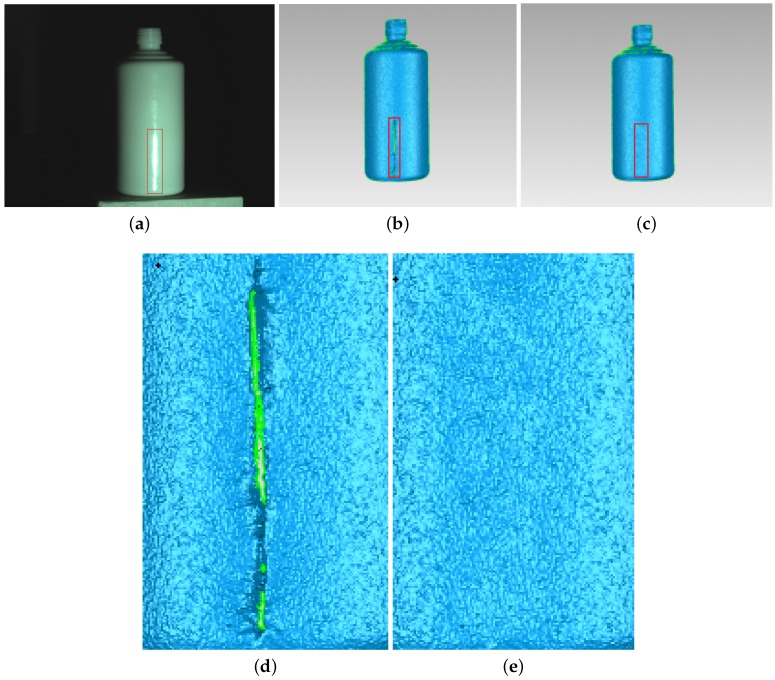
Comparison of the reconstruction of ceramic bottle before and after processing. (**a**) ceramic bottle; (**b**) before processing; (**c**) after processing; (**d**) enlarged view of red frame in (**b**); and (**e**) enlarged view of red frame in (**c**).

Figure 8 shows the ceramic bottle images projected by coded fringe pattern, in which there are obvious highlights. Figure 10b shows the reconstruction result without highlight removal, and the absence of information is caused due to specularities, as shown by the vacant areas in Figure 10d. Figure 9b shows the processed ceramic bottle with our method. As can be seen from Figure 9, the highlight region is significantly reduced. Figure 10c gives the three-dimensional reconstruction result of Figure 9, and the reconstructed effect is better than the effect in Figure 10b.

**Figure 11:**
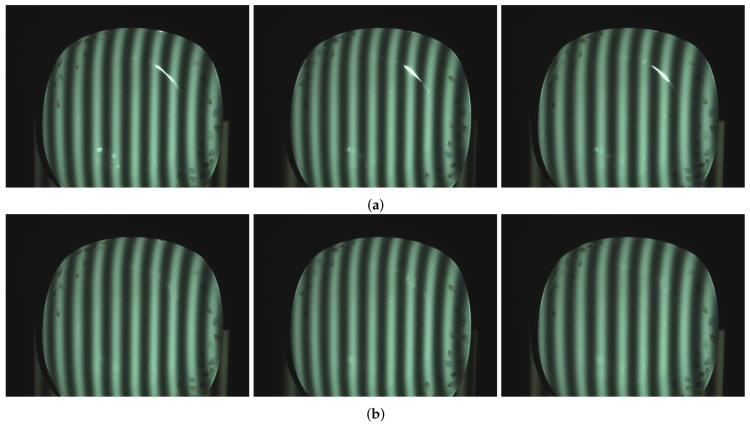
Comparison of ceramic plate before and after processing. (**a**) ceramic plate influenced by highlights; and (**b**) processed ceramic plate with our method.

**Figure 12:**
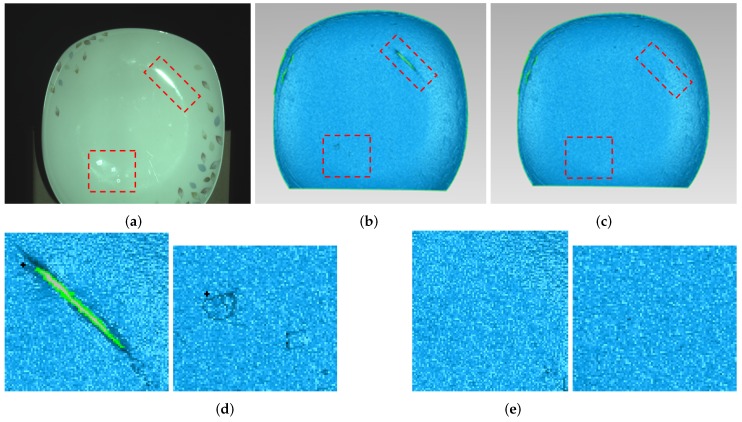
Comparison of the reconstruction of ceramic plate before and after processing. (**a**) ceramic plate; (**b**) before processing; (**c**) after processing; (**d**) enlarged view of red frame in (**b**); and (**e**) enlarged view of red frame in (**c**).

Figure 11 gives the comparison of the ceramic plate with the projected pattern before and after processing. As shown in Figure 11a, the upper and lower parts of the image have obvious highlight area. By using the proposed method, from Figure 11b, it is obvious that highlight regions disappear. Figure 12 shows the comparison result of the reconstruction before and after processing. As shown in Figure 12b, due to the loss of stripe information, there are vacant areas brought by highlights. Figure 12c shows reconstruction results processed by the proposed method, and we can see that, after processing, the plate is well reconstructed, the reconstructed surface is fine and smooth, and the highlight area disappears.

**Figure 13:**
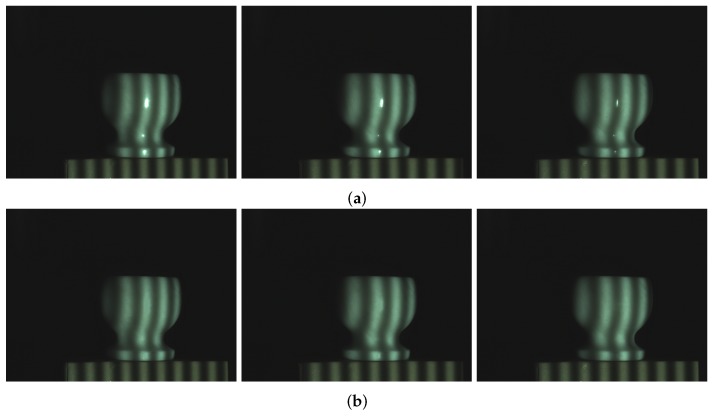
Comparison of marble pot before and after processing. (**a**) marble pot influenced by highlights; and the (**b**) processed marble pot with our method.

**Figure 14:**
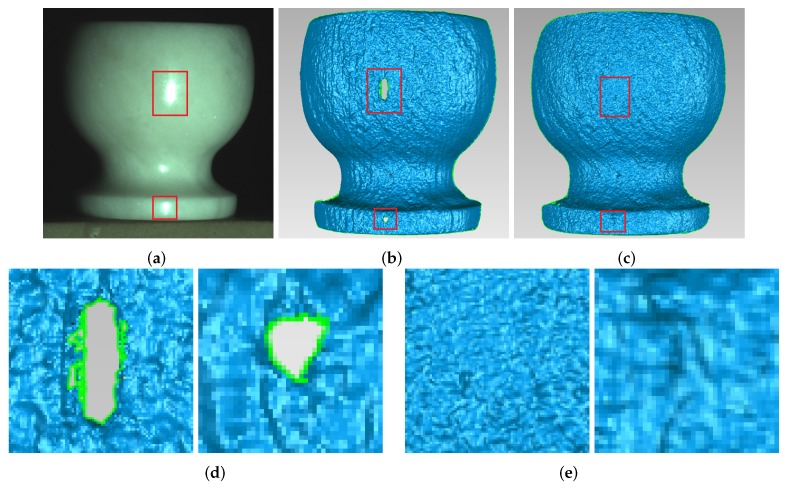
Comparison of the reconstruction of marble pot before and after processing. (**a**) marble pot; (**b**) before processing; (**c**) after processing; (**d**) enlarged view of red frame in (**b**); and (**e**) enlarged view of red frame in (**c**).

In order to test the effectiveness of this approach working with objects having different material, the marble pot was chosen as the measured object. Figure 14a shows the marble pot, of which the surface is slightly rougher than the ceramic surface. As shown in Figure 14a, the middle part and the base of the marble pot have obvious highlight areas. Figure 14b shows the reconstruction result without highlight removal, and the absence of information is caused by specularities, as shown by the vacant areas in Figure 14d. Figure 13 shows the comparison results of the marble pot before and after processing. Compared with Figure 13a, it is obviously that highlight phenomenon is eliminated in Figure 13b. Figure 14c shows reconstruction results processed by the proposed method, and we can see that, after processing, the marble pot is well reconstructed, and the highlight area disappears.

**Figure 15:**
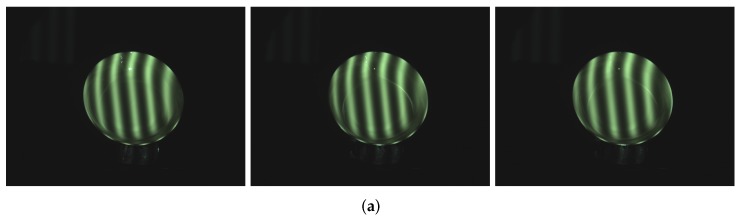

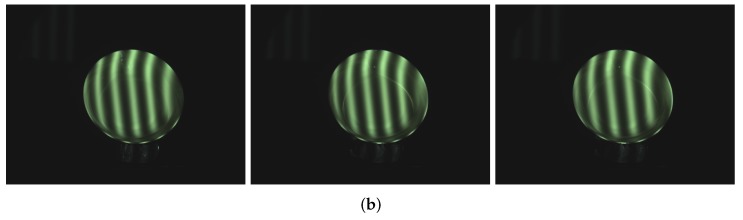
Comparison of yellow plate before and after processing. (**a**) yellow plate influenced by highlights; and (**b**) processed yellow plate with our method.

**Figure 16:**
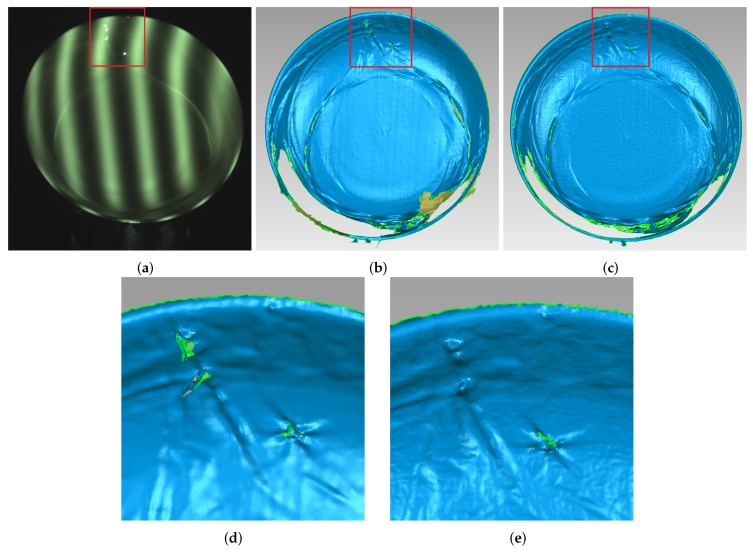
Comparison of the reconstruction of yellow plate before and after processing. (**a**) yellow plate; (**b**) before processing; (**c**) after processing; (**d**) enlarged view of red frame in (**b**); and (**e**) enlarged view of red frame in (**c**).

In order to test the effectiveness of this approach working with objects having different colors, the yellow ceramic plate was chosen as the measured object. Figure 15 gives the comparison of yellow ceramic plate with the projected pattern before and after processing. As shown in Figure 15a, the upper part of the yellow plate has an obvious highlight area. By using the proposed method, from Figure 15b, it is obvious that the highlight region disappears. Figure 16 shows the comparison result of the reconstruction before and after processing. As shown in Figure 16b, due to the loss of stripe information, there are vacant areas brought by the highlights. Figure 16c shows reconstruction results processed by the proposed method, and we can see that, after processing, the plate is well reconstructed, and the highlight area disappears.

**Figure 17:**
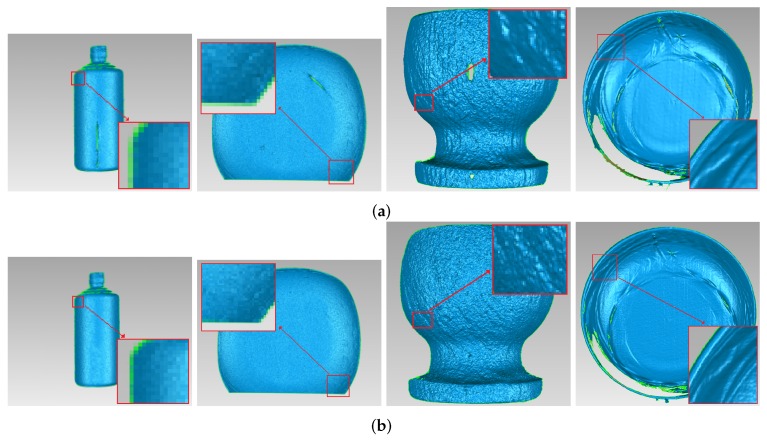
3D reconstruction results. (**a**) before processing; and (**b**) after processing.

In order to present 3D reconstruction results, as shown in Figure 17, we magnified the reconstruction surfaces. From Figure 17, we can see that ceramic plate, ceramic bottle, and marble pot are all vividly reconstructed. The magnified parts of the figure also prove that the details of these measured objects are clearly displayed and reconstructed surfaces are fine and smooth, which proves that the proposed approach is suitable for the object with highlight.

### 4.3. Quantitative Analysis

In order to more intuitively describe the proposed highlight removal method, Table 1 gives the number of highlight pixels before and after the processing. For the ceramic bottle, the number of highlight pixels decreased from 10,326 to 236 by about 43.8 times. For the ceramic plate, the number of highlight pixels decreased by 41.4 times from 5637 to 136. For the marble pot, the number of highlight pixels decreased by 33.0 times from 3365 to 102. For the yellow plate, the number of highlight pixels decreased by 10.1 times from 162 to 16.

### 4.4. Objective Performance Comparisons

**Figure 18:**
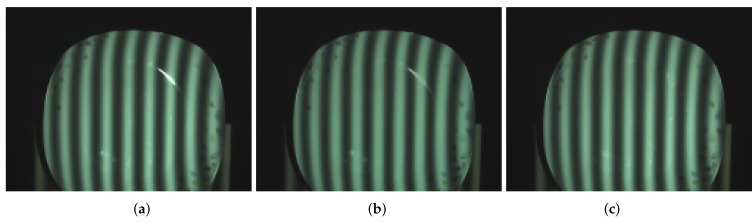
Comparison result between the proposed method and the color space conversion method. (**a**) the original image; (**b**) color space conversion method; and (**c**) proposed method.

To evaluate the performance of the proposed approach in the present paper, we compare it with the color space conversion method [29]. For removing high-intensity regions in a single image, the color space conversion method extracts the excessively illuminated regions and changes their brightness. Figure 18 is the comparison result between the proposed method and the color space conversion method, as shown in Figure 18b. The highlights have been weakened, but the overall brightness of the image has changed.

## 5. Conclusions

In a structured light 3D measurement field, when the object has a smooth surface, it can form a highlight area due to the specular reflection, and the distortion of the object will cause a measurement error. In this paper, for the purpose of removing highlight pixels effectively, we proposed a specular highlight removal method based on reflection component separation (RCS) and priority region filling theory, which does not depend on either polarization or image segmentation. Primarily, RCS theory is used to separate reflection components, i.e., determining the target highlight region. Then, priority region filling theory was used to restore the color information of the highlight region. Finally, we build up the system platform and implement 3D experiments on objects with strong reflecting surfaces like ceramic plates, bottles, marble pots and yellow plates.

Experimental results show that with the proposed approach, the highlight pixel numbers of the ceramic bottle, ceramic plate, marble pot and yellow plate are decreased by 43.8 times, 41.4 times, 33.0 times and 10.1 times, which proves that this method can effectively remove the specular pixels. Furthermore, 3D reconstruction results of surfaces show that the reconstructed surface is fine and smooth, and the detail features of the measured objects are clearly displayed, which proves that the proposed approach is suitable for objects with strong reflecting surfaces. Future work may include integrating prior knowledge and machine learning to restore much larger highlight regions.

## Figures and Tables

**Table 1:** The number of highlight pixels before and after the processing.

Measured Object	The Number of Highlight Pixels	Total Number of Pixels in Red Frame	Percentage	Reduction
Original image of ceramic bottle	10,326	88,660	11.6%	43.8
Processed image of ceramic bottle	236		0.27%	
original image of ceramic plate	5637	37492	15.0%	41.4
Processed image of ceramic plate	136		0.36%	
original image of marble pot	3365	62,944	5.3%	33.0
Processed image of marble pot	102		0.16%	
original image of yellow plate	162	9183	1.76%	10.1
Processed image of yellow plate	16		0.17%	

## References

[1] Bui L.Q., Lee S. (2013). Boundary Inheritance Codec for high-accuracy structured light three-dimensional reconstruction with comparative performance evaluation. Appl. Opt..

[2] Chen F., Brown G.M., Song M. (2000). Overview of three dimensional shape measurement using optical methods. Opt. Eng..

[3] Blais F. (2004). Review of 20 years of range sensor development. Electron Imaging.

[4] Zhang Z.H. (2012). Review of single-shot 3D shape measurement by phase calculation-based fringe projection techniques. Opt. Lasers Eng..

[5] Nayar S.K., Gupta M. Diffuse Structured Light. Proceedings of the IEEE International Conference on Computational Photography.

[6] Shafer S. (1985). Using color to separate reflection components. Color Res. Appl..

[7] Klinker G.J., Shafer S.A., Kanade T. (1988). The measurement of highlights in color images. Int. J. Comput. Vis..

[8] Mallick S.P., Zickler T.E., Kriegman D.J., Belhumeur P.N. Beyond lambert: Reconstructing specular surfaces using color. Proceedings of the 2005 IEEE Computer Society Conference on Computer Vision and Pattern Recognition.

[9] Kokku R., Brooksby G. (2005). Improving 3D surface measurement accuracy on metallic surfaces. Proc. SPIE.

[10] Yang Y.M., Fan J.Z., Zhao J. (2010). Preprocessing for highly reflective surface defect image. Opt. Precis. Eng..

[11] Chai Y.T., Wang Z., Gao J.M., Huang J.H. (2013). Highlight Removal Based on Frequency-Domain Filtering. Laser Optoelectron. Prog..

[12] Wolff L., Boult T. (1991). Constraining object features using polarization reflectance model. IEEE Trans. Pattern Anal. Mach. Intell..

[13] Nayar S., Fang X., Boult T. Removal of Specularities using Color and Polarization. Proceedings of the IEEE Computer Society Conference on Computer Vision and Pattern Recognition.

[14] Sohn B.J., Lee S. (2013). Analytical relationship between polarized reflectivities on the specular surface. Int. J. Remote Sens..

[15] Tsuru T. (2013). Tilt-ellipsometry of object surface by specular reflection for three-dimensional shape Measurement. Opt. Express.

[16] Feris R., Raskar R., Tan K.H., Turk M. (2004). Non-photorealistic camera: Depth edge detection and stylized rendering using multi-flash imaging. ACM Trans. Graph..

[17] Liu Y.K., Su X.Y., Wu Q.Y. (2006). Three Dimensional Shape Measurement for Specular Surface Based on Fringe Reflection. Acta Opt. Sin..

[18] Qian X.P., Harding K.G. (2003). Computational approach for optimal sensor setup. Opt. Eng..

[19] Hu Q., Harding K.G., Du X., Hamilton D. (2005). Shiny parts measurement using color separation. Proc. SPIE.

[20] Sato Y., Ikeuchi K. (2001). Temporal-color space analysis of reflection. J. Opt. Soc. Am. A.

[21] Zheng J.Y., Fukagawa Y., Abe N. (1997). 3D surface estimation and model construction from specular motion in image sequences. IEEE Trans. Pattern Anal. Mach. Intell..

[22] Liu G.H., Liu X.Y., Feng Q.Y. (2011). 3D shape measurement of object with high dynamic range of objects with high dynamic range of surface reflectivity. Appl. Opt..

[23] Asundi A.K. (1993). Moiré methods using computer-generated gratings. Opt. Eng..

[24] Jiang Y.Z. (2000). Acquiring a Complete 3D Model from Specular Motion under the Illumination of Circular-Shaped Light Sources. IEEE Trans. Pattern Anal. Mach. Intell..

[25] Guo H.W., Tao T. (2007). Specular surface measurement by using a moving diffusive structured light source. Proc. SPIE.

[26] Sills K., Bone G.M., Capson D. (2014). Defect identification on specular machined surfaces. Mach. Vis. Appl..

[27] Tan R.T., Ikeuchi K. (2005). Separating Reflection Components of Textured Surfaces Using a Single Image. IEEE Trans. Pattern Anal. Mach. Intell..

[28] Criminisi A., Perez P., Toyama K. Object removal by exemplar-based inpainting. Proceedings of the 2003 IEEE Computer Society Conference on Computer Vision and Pattern Recognition.

[29] Wang C.Q., Zhu F.W. (2007). Remving Highly Illuminated Regions from a Single Image. J. Shanghai University.

